# Decreased Cancer Consultations in the COVID-19 Era: A Concern for Delay in Early Cancer Diagnosis in India

**DOI:** 10.1200/GO.21.00030

**Published:** 2021-03-24

**Authors:** Vishal Rao, Gururaj Arakeri, Anand Subash, BS Ajaikumar, Ramesh Patil, Beverly Hale, Rui Amaral Mendes

**Affiliations:** Vishal Rao, MS; Gururaj Arakeri, MDS, PhD;Anand Subash, MS; and BS Ajaikumar, MD, Department of Head and Neck Oncology, HealthCare Global Cancer Hospital, Bengaluru, Karnataka, India; Ramesh Patil, MSc, Ashwini Rural Medical College Hospital and Research Center, Solapur, Maharashtra India; Beverly Hale, MSc, University of Chichester, Chichester, West Sussex, United Kingdom; and Rui Amaral Mendes, DMD, PhD, Department of Oral and Maxillofacial Medicine and Diagnostic Sciences, Case Western Reserve University, Cleveland, OH; CINTESIS—Centre for Health Technology and Services Research, Porto, Portugal

The COVID-19 pandemic has caused unprecedented impact across the globe, severely affecting the national health systems, as well as mobility and commuting. Several key health issues are inadvertently being overlooked, bearing impact on mortality and other outcomes, particularly for patients with cancer.

The recent global collaborative study that addressed the impact of the SARS-Cov-2 surge on cancer care not only reported on its varying magnitude among centers worldwide but also further called for additional research to assess this impact at the patient level.^1^

We too find this to be a major problem, as modeling studies herald a general concern over COVID-19–related delays in peri-management of oncological patients, leading to an increased loss of life and life-years linked to patient age and tumor type^2-5^ with reports of an overall 55% decrease in referrals, whereas the diagnostic yield increased from 2.9% in January to 8.06% in April.^2^

Moreover, the pandemic appears to strengthen the impact of pre-existing social disparities, making it central that we address the social determinants of health to provide equitable, high-quality care for patients with head and neck cancer,^6^ therefore overcoming the reported impact of COVID-19 on healthcare and socioeconomic systems in Asia Pacific.^7^

As algorithms have been suggested to stratify patients requiring head and neck cancer surgery in the COVID-19 era,^8^ it is paramount that we do not lose sight of the severe upstream impact leading to delayed diagnosis or follow-up of survivors. In fact, this inordinate delay in the examination and diagnosis of this ailment has severely affected mortality and other outcomes, particularly for patients with cancer.^3,9^

Still, early diagnosis of cancer, although critical in every respect, is mostly dependent on the patient's willingness to consult physicians and attend routine examinations.^9^

Overall, the pandemic has forced a majority of patients to approach healthcare centers only for emergency treatments, thereby reducing the frequency of early clinical examinations and hence detection of cancer.

After imposing a nationwide lockdown for two months, India underwent three phases of unlocks: unlock 1 from June 1 to June 30, 2020; unlock 2 from July 1 to July 31, 2020; and unlock 3 August 1 to August 31, 2020.^10^

**FIG 1:**
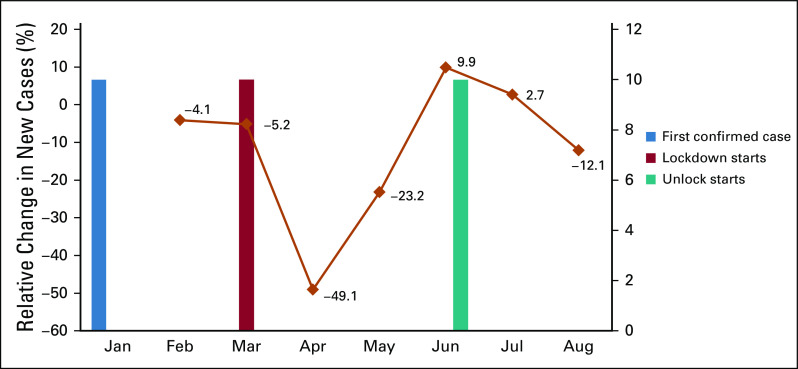
Number of cancer consultations by month in India (data from 24 branches of HCG cancer center) in the period between January 1, 2020, and August 30, 2020.

The lockdown or unlock stipulations have significantly affected the Indian cancer care system. As an example, several of our 24 oncology centers across India (HCG Cancer Hospitals) witnessed a cumulative drop of new consultations (49.1%) immediately after the lockdowns were imposed (Fig 1). Interestingly, we noticed a gradual rise (9.9%) in new consultations once the extension of lockdown was announced. However, once the unlock was enforced, there was a progressive decline in new consultations (12.1%).

In hindsight, the reasons for this pattern can be gathered with reasonable accuracy. Initially, in the early phase of the pandemic, the fear and panic linked with announcement of lockdown and virus spread might have forced patient to postpone their heath checkups. With continuous imposition of restrictions and prevailing uncertainties, the patients summoned the courage to visit the hospitals and to get examined. A matter of grave concern, however, is the progressive gradual drop of new consultations (12.1%) even after the announcement of the unlock.

Several factors could possibly explain the drop in new consultations after the lockdown: (1) individuals with nonspecific symptoms of cancer might have had some anxiety about contracting COVID-19 during commuting, as also within a healthcare setting; (2) many patients may be unsure about the adequacy and capacity of essential non-COVID-19–related healthcare services; (3) the misconception that COVID-19 testing is mandatory for undertaking any treatment at hospital might have dissuaded patients from visiting the health setting; (4) many government and private cancer screening programs have been temporarily halted or working at very slow pace even after the unlock; and (5) the prevailing stay-at-home orders in densely populated areas affect segments of the population known to share both an increased risk for COVID-19 and for head and neck cancer.

The disturbing findings of fewer consultations even after the announcement of unlock warrant immediate attention. If the delay in early consultations continues unabated, it may negatively influence early cancer detection, resulting in increasing tumor burden and a likely upstaging of TNM classification, negatively affecting outcomes and overall quality of life of patients with head and neck cancer.

As the COVID-19 pandemic appears still far from declining, the healthcare system demands immediate attention to quickly help restore it to normalcy. As suggested by Dinmohamed et al,^9^ a couple of key measures are the need of the hour: (1) individuals with suspicious symptoms need to be proactively encouraged to consult their general practitioners and (2) the medical fraternity should provide timely support and guidance to policymakers for designing and delivering public awareness programs.
